# Effectiveness of Heel-Raise-Lower Exercise after Transcutaneous Electrical Nerve Stimulation in Patients with Stroke: A Randomized Controlled Study

**DOI:** 10.3390/jcm9113532

**Published:** 2020-10-31

**Authors:** Kyoung-sim Jung, Jin-hwa Jung, Tae-sung In, Hwi-young Cho

**Affiliations:** 1 Department of Physical Therapy, Gimcheon University, Gimcheon 39528, Korea; 20190022@gimcheon.ac.kr; 2 Department of Occupational Therapy, Semyung University, Jecheon 27136, Korea; otsalt@semyung.ac.kr; 3 Department of Physical Therapy, Gachon University, Incheon 21936, Korea

**Keywords:** electrical stimulation, plantar flexor, spasticity, stroke

## Abstract

Objective: This study was conducted to investigate the effect of the heel-raise-lower exercise on spasticity, strength, and gait speed after the application of 30 min of transcutaneous electrical nerve stimulation (TENS) in patients with stroke. Methods: The participants were randomly divided into the TENS group and the placebo group, with 20 participants assigned to each group. In the TENS group, heel-raise-lower exercise was performed after applying TENS for six weeks. The placebo group was trained in the same manner for the same amount of time but without electrical stimulation. The spasticity of the ankle plantar flexors was measured using the composite spasticity score. A handheld dynamometer and a 10-m walk test were used to evaluate muscle strength and gait speed, respectively. Results: Spasticity was significantly more improved in the TENS group (mean change −2.0 ± 1.1) than in the placebo group (mean change −0.4 ± 0.9) (*p* < 0.05). Similarly, muscle strength was significantly more improved in the TENS group (6.4 ± 3.3 kg) than in the placebo group (4.5 ± 1.6 kg) (*p* < 0.05). Moreover, participants assigned to the TENS group showed a significant greater improvement in gait speed than those in the placebo group (mean change −5.3 ± 1.4 s vs. −2.7 ± 1.2 s). Conclusions: These findings show the benefits of heel-raise-lower exercise after TENS for functional recovery in patients with stroke.

## 1. Introduction

Patients with stroke experience gait disorders due to various causes, including sensory impairment and muscle weakness, spasticity, etc., and the primary goal of rehabilitation is to regain independent walking [1,2,3]. The ankle plantar flexors tightly contract to maintain a standing posture [4] and contribute to postural control through anticipatory contractions before changes in the center of mass [5,6,7]. The plantar flexors in the ankle create most of the energy required for forward propulsion of body mass during walking [8,9]. The strength of the ankle plantar flexors on the paretic side is related to the gait speed of patients with stroke [3,10,11]. 

The heel-raise exercise is commonly applied in clinical settings to strengthen the plantar flexors in the standing position and can be performed without the use of any equipment [12,13]. Fujiwara et al. [14] reported a significant increase in the strength and thickness of the plantar flexors and a decrease in postural sway in elderly subjects after eight weeks of heel-raise exercise. Another study reported a significant increase in plantar flexor power, walking speed, and cadence in stroke patients who performed a heel-raise exercise using a block to achieve dorsiflexion of the forefoot compared with those who performed the same exercise on a flat surface. The authors explained that the use of a block enables concentric and eccentric contractions of the plantar flexors over the full range of motion of the ankle [13]. However, dorsiflexion is difficult in patients with stroke who have plantar flexor spasticity [15]. Eccentric contractions promote the activation of paretic muscles [16]. Plantar flexor spasticity must be controlled so that the muscles can sufficiently stretch and undergo eccentric contractions.

Transcutaneous electrical nerve stimulation (TENS) has recently been used to improve spasticity caused by neurological disorders. Several meta-analyses have reported that TENS effectively reduces spasticity in stroke patients [17,18,19]. Mahmood et al. [18] reported that TENS effectively healed spasticity when applied on the nerves or muscle belly for > 30 min. Jung et al. [20] reported that TENS effectively treated spasticity after sit-to-stand training, and significantly improved lower-extremity muscle strength and postural sway compared with placebo.

The heel-raise exercise is commonly applied in clinical settings. However, studies examining the effect of the exercise in stroke patients are limited and no study to date has examined whether the heel-raise exercise is more effective when combined with TENS. Therefore, the purpose of this study was to compare the effects of heel-raise-lower exercise with and without electrical stimulation on spasticity, muscle strength, and gait speed in stroke patients.

## 2. Experimental Section

### 2.1. Participants

The participants of this study were 40 patients who were admitted to K Hospital in South Korea. The inclusion criteria were as follows: (1) A diagnosis of stroke, (2) first episode of unilateral stroke with hemiparalysis caused by hemicerebrum damage, (3) subacute patients with an onset period of less than 12 months, (4) ability to communicate, (5) ability to walk 10 m independently, (6) moderate to severe spasticity of the paretic ankle (composite spasticity score ≥ 10) [21,22,23], (7) a medically stable status, (8) no history of peroneal nerve lesions, (9) no neglect and sensory loss [24], (10) no orthopedic disease that can influence walking, (11) who have not previously received TENS, and (12) no contraindications to TENS. The general characteristics of the participants are shown in Table 1.

### 2.2. Protocol

This study employed a pretest-posttest control group design in which the participants were divided, according to the intervention method, into the TENS group and placebo group. All participants were assessed for spasticity, muscle strength, and gait speed before and after intervention.

In this study, the double-blind method was designed to increase the reliability of evaluation. Three physical therapists with more than 5 years of clinical experience and a master’s degree or higher performed measurements or interventions under blinded conditions. In addition, subjects participated in the study without knowing any information about the group to which they belong and the interventions to be received. All participants provided written informed consent and the ethics committee of Gachon University approved the study (1044396-202006-HR-114-01). The trial was registered under trial registration no. KCT0005217.

### 2.3. Intervention

In the TENS group, a TENS machine (TENS-7000; Koalaty Products Inc., Tampa, FL, USA) was used to provide electrical stimulation for 30 min before the heel-raise-lower exercise training. The electrode was attached to the affected peroneal nerve. The TENS group received stimulation at twice the intensity of producing a tingling sensation, to the extent that muscle contractions did not occur. The pulse width and frequency were set to 200 µs and 100 Hz, respectively. The participants were instructed to immediately report any involuntary muscle contraction or discomfort.

In participants in the placebo group, the electrode was attached to the same location without electrical stimulation. The researcher showed the subject to turn on the TENS apparatus and gave the subject very fine electrical stimulation that the subject could feel. When the subject expressed that electrical stimulation was felt, the researcher turned off the power of TENS apparatus while hiding the TENS in the box, and explained that a microcurrent of TENS was being applied to the subject. 

Participants in both groups placed their forefeet on a block with a height that allowed heel contact with the floor according to the extensibility of the plantar flexors during the heel-raise-lower exercise. The participants performed repeated concentric and eccentric contractions of the plantar flexors by raising both heels as high as possible and lowering them slowly for approximately 2 s. To promote the contraction of the affected plantar flexors, the participants were instructed to symmetrically support their weight during exercise. Since the speed is different for each patient, the amount of exercise was set with the goal of repeating 100 times rather than time in order to equalize the amount of exercise [13]. Training was conducted 5 times a week for 6 weeks. At the beginning of training, most of the subjects started training on a block with a height of 5 cm, and trained by gradually increasing the height of the block. 

### 2.4. Outcome Measurements

The strength of the plantar flexors was measured using a handheld dynamometer (Model 01163; Lafayette Inc., Lafayette, IN, USA). To measure the muscle strength of the ankle plantar flexors, the participants were asked to perform plantar flexion against the dynamometer, with the hips and knees extended while lying on a mat. Measurements were performed three times and the average value was used. This measurement method has been reported to have good intra-rater reliability and inter-rater reliability for neurological patients [25].

A composite spasticity score consisting of Achilles tendon jerk, passive ankle dorsiflexion, and ankle clonus was used to evaluate the stiffness of the plantar flexors [15,24,26]. A score of ≤ 9 means mild spasticity; a score of 10–12 means moderate spasticity; and a score of 13–16 means severe spasticity [27].

A 10-m walk test was used to measure the gait speed of the participants. This test measures the time taken to walk 10 m. This test has been reported to have high intra-rater reliability and inter-rater reliability (r = 0.89–1.00) [28].

### 2.5. Data Analysis

SPSS 21.0 was used for statistical analysis. The Shapiro–Wilk test was used to evaluate the normality of the variables. The independent *t*-test and chi-square test were used to compare the baseline characteristics of the two groups of continuous and categorical variables. A paired *t*-test was performed to examine the changes within a group in spasticity, strength, and gait speed. An independent *t*-test was used to determine any significant differences between the two groups in the amount of change in spasticity, strength, and gait speed before and after 6 weeks of training. The significance level was set at *p* < 0.05. 

## 3. Results

### 3.1. General Characteristics of Participants

All general characteristics in both groups were homogeneous (Table 1).

### 3.2. Changes in Spasticity

The TENS group showed a greater degree of improvement in spasticity than the placebo group (mean score change, −2.0 ± 1.1 vs. −0.4 ± 0.9) (*p* < 0.05) (Table 2).

### 3.3. Changes in Muscle Strength

Significantly greater improvements in muscle strength were observed in the TENS group (mean change, 6.4 ± 3.3 kg) than in the placebo group (mean change, 4.5 ± 1.6 kg) (*p* < 0.05) (Table 3).

### 3.4. Changes in Gait Speed

Significantly greater improvements in gait speed were observed in the TENS group than in the placebo group (mean change, −5.3 ± 1.4 s vs. −2.7 ± 1.2 s) (*p* < 0.05) (Table 4).

We instructed the subjects and therapists that participated in the study to report any side effects from the application of TENS to the subjects. Symptoms such as redness, itchiness, pain, abnormal sensations, and other dermatitis of application due to the attachment of TENS were educated as side effects. Fortunately, none of the subjects showed these side effects during the study period. Through this, we suggested that TENS has a very low risk of side effects and can be effectively used to improve the spasticity of stroke patients in clinic.

## 4. Discussion

This study examined the effectiveness of the heel-raise-lower exercise on plantar spasticity after the application of TENS on the peroneal nerve in patients with stroke. Plantar flexor spasticity was significantly reduced in the TENS group compared with the placebo group. Several studies have reported reduced spasticity without any adverse effects after the application of 30 min of TENS on the peroneus nerve [20,29] and muscle belly of the plantar flexors [30,31,32] in patients with stroke. Other studies have shown that TENS relieves spasticity in muscles through presynaptic inhibitory effects [22,33,34] and a reduction of antagonist muscle co-contractions [35].

The plantar flexors create maximum torque when the ankle is slightly dorsiflexed [36]. Lee et al. reported a significant increase in plantar flexor strength in patients with stroke who performed heel-raise-lower exercise using a 5-cm-tall block to achieve dorsiflexion of the ankle joint and the forefoot compared with patients who performed the same exercise on a flat surface. They explained that repeating eccentric contractions of the plantar flexors in a large range of motion further stretched the calf muscle. Repeated eccentric contractions provide afferent input through muscle spindles and Golgi tendon organs, thereby allowing the calf muscle to stretch [37]. However, stabilizing the foot on the ground is difficult in patients with stroke and spasticity owing to limited dorsiflexion [38]. In the present study, a block of appropriate height was used in each patient with varying plantar flexor extensibility to ensure heel contact with the ground. The average height of blocks used by the TENS group and placebo group at week 1 was 5.8 ± 1.6 and 6.2 ± 1.8 cm, respectively, and the average height of blocks used at week 6 was 8.4 ± 3.1 and 7.9 ± 2.5 cm, respectively. At the beginning of training, most subjects used blocks of 5 cm. However, at the 6th week, subjects who were close to half of the TENS group used blocks of 10 cm. This is because TENS alleviated the stiffness of the plantar flexors, so it was possibly the result of increased extensibility. TENS stabilizes heel contact by reducing plantar flexor spasticity [20] and weight bearing by reducing the resistance against anterior rotation of the tibia over the foot [21,23]. The significant improvement in plantar flexor strength in the TENS group was attributed to improved efficiency in eccentric contractions due to spasticity reduction. As eccentric contractions occur during heel contact, accurate afferent input and additional feedback with respect to the target angle may have further promoted muscle activation.

Furthermore, this study investigated the effect of the heel-raise exercise after TENS on gait speed. The TENS group showed significant improvements compared with the placebo group. The calf muscle is important in anticipatory postural adjustment [6]. A study reported a significant increase in calf muscle thickness and a decrease in the postural sway of elderly subjects after heel-raise exercise [14]. Furthermore, one of the main factors affecting walking speed is muscle strength [39]. The plantar flexors reach peak power and generate propulsive power during the push-off phase of gait [10,40,41]. Brincks and Nielsen [3] reported increased plantar flexor strength in patients with stroke after robotic gait training, and revealed that increased muscle strength was significantly associated with improved gait speed. In the present study, the significant improvement in the gait speed of the TENS group was attributed to improved plantar flexor strength, which subsequently improved postural balance.

In this study, the heel-raise exercise combined with TENS resulted in significant improvements in plantar flexor spasticity, plantar flexor strength, and gait speed in patients with stroke. However, there are some limitations in this study. The results of this study cannot be generalized because the sample size is small and the study was conducted only on patients with subacute stroke. Furthermore, it was not confirmed how the training method of this study affects proprioception or gait patterns including gait symmetry. Future studies investigating the effect of heel-raise exercise and TENS on proprioception or qualitative factors of gait, such as ankle movements and knee joint angles in different gait phases, in patients with stroke are needed.

## 5. Conclusions

The results of this study proved that heel-raise-lower exercise after the application of TENS was beneficial for the management of spasticity, muscle strength, and gait function in stroke patients with spasticity in the plantar flexor muscles of the ankle.

## Figures and Tables

**Table 1:** Common and clinical characteristics of the participants (N = 40).

Variables	TENS Group (n = 20)	Placebo Group (n = 20)	*p*-Value ^a^
Sex			
Male	14	12	0.741 ^a^
Female	6	8	
Age (years)	53.1 ± 7.9 ^a^	52.7 ± 11.5	0.886 ^b^
Weight (kg)	63.2 ± 9.0	65.9 ± 8.2	0.325 ^b^
Height (cm)	165.5 ± 9.4	166.7 ± 10.5	0.695 ^b^
Stroke type			
Ischemic	13	11	0.748 ^a^
Hemorrhagic	7	9	
Post-stroke duration (months)	6.8 ± 2.5	7.0 ± 2.6	0.758 ^b^

Values are expressed as mean ± standard deviation. ^a^ Chi-square test. ^b^ Independent *t*-test. Transcutaneous electrical nerve stimulation (TENS).

**Table 2:** Changes in spasticity of the study participants (N = 40).

	TENS Group			Placebo Group		Difference	*p*-Value
	Pretest	Posttest	Difference	Pretest	Posttest		
CSS (score)	11.5 ± 1.6	9.5 ± 1.9 *	−2.0 ± 1.1	11.9 ± 2.1	11.5 ± 2.3	−0.4 ± 0.9	0.000

CSS, composite spasticity score. * Significant difference between pretest and posttest (*p* < 0.05).

**Table 3:** Changes in muscle strength of the study participants (N = 40).

	TENS Group			Placebo Group		Difference	*p*-Value
	Pretest	Posttest	Difference	Pretest	Posttest		
Muscle strength (kg)	12.0 ± 2.0	18.4 ± 3.5 *	6.4 ± 3.3	11.6 ± 2.0	16.1 ± 1.9	4.5 ± 1.6	0.000

* Significant difference between pretest and posttest (*p* < 0.05).

**Table 4:** Changes in gait speed of the study participants (N = 40).

	TENS Group			Placebo Group		Difference	*p*-Value
	Pretest	Posttest	Difference	Pretest	Posttest		
10MWT time (s)	24.7 ± 4.0	19.4 ± 3.3 *	−5.3 ± 1.4	25.2 ± 4.8	22.5 ± 5.0 *	−2.7 ± 1.2	0.000

10 MWT, 10 m walk test. * Significant difference between pretest and posttest (*p* < 0.05).
